# Prevalence of vaginal laxity in primiparous women six months after birth

**DOI:** 10.61622/rbgo/2025rbgo61

**Published:** 2025-07-15

**Authors:** Marina Resende Godoy, Gláucia Miranda Varella Pereira, Clara Vale Viegas, Marilene Vale de Castro Monteiro

**Affiliations:** 1 Universidade Federal de Minas Gerais Faculdade de Medicina Departamento de Ginecologia e Obstetrícia Belo Horizonte MG Brazil Departamento de Ginecologia e Obstetrícia, Faculdade de Medicina, Universidade Federal de Minas Gerais, Belo Horizonte, MG, Brazil.; 2 Universidade de Campinas Faculdade de Ciências Médicas Departamento de Ginecologia e Obstetrícia Campinas SP Brazil Departamento de Ginecologia e Obstetrícia, Faculdade de Ciências Médicas, Universidade de Campinas, Campinas, SP, Brazil.; 3 Faculdade de Ciências Médicas de Minas Gerais Belo Horizonte MG Brazil Faculdade de Ciências Médicas de Minas Gerais, Belo Horizonte, MG, Brazil.

**Keywords:** Urinary incontinence, Postpartum period, Constipation, Surveys and questionnaires, Vaginal laxity

## Abstract

**Objective:**

To assess the prevalence of VL in primiparous women undergoing vaginal birth or caesarean section; and its association with obstetric, urinary, intestinal and sexual factors for its occurrence.

**Methods:**

This is a cross-sectional study carried out between July 2021 and January 2023. Primiparous women who underwent vaginal birth or caesarean section without complaints of VL during pregnancy were included. Clinical and obstetric data were collected and participants completed questionnaires on the impact of urinary incontinence (ICIQ-SF), vaginal symptoms (ICIQ-VS) and sexual distress (FSDS-R) at recruitment and six-months postpartum. Univariate and multivariate logistic regression was performed, considering VL as the outcome and p=0.05.

**Results:**

One hundred participants were included for data analysis. The prevalence of VL was 8%. In the univariate analysis, SUI, urgency urinary incontinence (UUI), coital incontinence, constipation and the ICIQ-VS and ICIQ-SF scores were associated with VL. The ICIQ-VS, FSDS-R and ICIQ-SF scores increased the risk of VL by one-fold. However, only UUI (OR 10.50(CI 95% 1.90-58.10), coital incontinence (OR 42.00(CI 95% 3.11-566.38), and ICIQ-VS (vaginal symptoms OR 1.32(CI 95% 1.05-1.66) and ICIQ-SF (OR 1.25(CI 95% 1.02-1.54), scores remained associated with VL in multivariate-analysis.

**Conclusion:**

The prevalence of VL in primiparous women was lower than that reported in other studies and showed an association with the occurrence of vaginal symptoms, UUI and coital incontinence, six- months postpartum.

## Introduction

Vaginal laxity (VL) is a common postpartum complaint,^(1,2)^ and is characterized by excessive vaginal looseness perceived by the patient.^(3)^

According to the literature, its prevalence can vary between 2% and 48%.^(1,4,5)^ VL appears to be associated with changes in the integrity of vaginal and muscular tissue due to pregnancy, childbirth and ageing, resulting in greater distension of the pelvic floor muscles and opening of the vaginal introitus.^(6,7)^ Excessive distension of the pelvic floor muscles contributes to the occurrence of microtraumas and avulsion of the levator ani muscle, leading to an increase in the size of the genital hiatus.^(8)^ Such changes may result in reduced sensation during penetration, orgasmic dysfunction, and reduced sexual satisfaction.^(5,7,9)^ Reduced sensation during sexual intercourse may be related to damage to the perineal body, vaginal introitus, nerves, and connective tissue.^(5)^

VL is common in young women and after vaginal birth.^(1,4)^ Caesarean section appears to be a protective factor for the symptom.^(4)^ The symptom of VL pre-existing during pregnancy does not seem to worsen after birth, but women who had a vaginal birth had a higher prevalence of VL compared to those who had a caesarean section.^(10)^ When compared to nulliparous women, primiparous women were five times more likely to report VL and women who had three or more vaginal births were seven times more likely to report VL.^(5,11)^

Although there is currently a growing interest in treatment options for VL, its pathophysiology is still poorly understood.^(12–14)^ According to 83% of the 416 physicians members of the International Urogynecological Association (IUGA) surveyed, the symptom of VL is underreported and presents itself as an uncomfortable condition that negatively affects the sexual life and affective relationships of their patients.^(14)^

Considering that the mechanism of occurrence of VL is little studied, further studies are needed on its association with the type of delivery and the first delivery. It appears that complaints of VL are more prevalent among women who have experienced vaginal childbirth, with the first delivery being the primary contributing factor. This observation aligns with previous research indicating that the most significant adverse effects on the pelvic floor occur during the first vaginal birth, while subsequent deliveries have a comparatively lesser impact.^(15)^ A study examined the influence of first and second deliveries on levator ani muscle (LAM) avulsion and its association with symptoms of pelvic floor dysfunction. Their findings revealed that the first delivery poses the greatest risk for LAM avulsion, whereas the second may exacerbate existing injuries without causing new avulsions.^(16)^ To date, there are no studies that address VL exclusively in primiparous women. Thus, the objective of this study was to assess the prevalence of VL in primiparous women undergoing vaginal or caesarean birth; and its association with risk factors (clinical, obstetric), in addition to urinary, intestinal and sexual symptoms for its occurrence at six months post-partum.

## Methods

This is a cross-sectional observational study, carried out between July 26, 2021, and January 31, 2023, at a tertiary hospital. We followed the STROBE recommendations for observational studies.^(17)^

Primiparous women, aged 18 years or older, who retrospectively reported an absence of symptoms related to vaginal laxity during pregnancy in the immediate postpartum period, and who underwent either vaginal delivery or cesarean section between July 2021 and January 2023, were included in the study. Participant selection was conducted weekly in the maternity ward. The absence of VL was considered when participants responded negatively to a direct question regarding their perception of VL (yes/no) and negative response to question 4a (Do you feel that your vagina is very loose or lose?) from the International Consultation on Incontinence Questionnaire - Vaginal Symptoms (ICIQ-VS) regarding the gestational period.^(18)^ Women were excluded if they reported VL during pregnancy, had a history of vaginal or caesarean birth, underwent vaginal surgery before pregnancy, had a gestational age of less than 34 weeks, had neurological disorders, experienced cognitive impairments or were unable to read or understand Brazilian Portuguese, or failed to respond to the questionnaires six months postpartum.

Participants who met the inclusion criteria were approached in person by a single researcher in the hospital room during the immediate postpartum period until up to 10 days after delivery. The researcher ensured that all participants could answer the questions accurately. Sociodemographic, clinical, and obstetric data were collected from medical records. Urinary, vaginal, and sexual symptoms were assessed using self-administered questionnaires validated for Brazilian Portuguese. These questionnaires were completed in person in the hospital room during the immediate postpartum period, gathering responses about the gestational period as baseline assessment. The six-month assessment was conducted via telephone or email six months after delivery.

The *International Consultation on Incontinence Questionnaire - Short Form* (ICIQ-SF) is a questionnaire that qualifies urinary loss and the impact of urinary incontinence (UI) on quality of life with a score ranging from 0 to 21. A score of zero means no urinary loss and no impact on quality of life.^(19)^ The ICIQ-VS assesses the severity of vaginal symptoms, the impact on sexual function and quality of life. The higher the score, the worse the severity of the symptoms.^(18)^ The *Female Sexual Distress Scale-Revised* (FSDS-R) is a questionnaire that aims to assess sexual distress with a maximum score of 53 and the higher the total score, the greater the sexual distress.^(2)^

To calculate the sample size, the proportion estimate was used in a descriptive study^(1)^ with a categorical qualitative variable, setting the alpha significance level or type I error at 5% (alpha = 0.05) (or 95% confidence interval), the sampling error at 10% (d = 0.10) and the sample power (1-beta) at 80%. A sample size of n = 109 participants was obtained for the present study. The sample calculation was performed by the SAS System for Windows (Statistical Analysis System), version 9.4. SAS Institute Inc, 2002-2012, Cary, NC, USA.

Continuous variables were presented as mean, standard deviation, and median, with minimum and maximum values reported. Categorical variables were presented according to their absolute and relative frequency. To estimate the association between the predictor variables and the outcomes of interest, the Chi-Square test was used for categorical variables and the Mann-Whitney test for continuous variables. Univariate and multivariate logistic regression models were used to estimate cofactor-adjusted odds ratios with 95% confidence intervals. In all statistical calculations, the significance level was 0.05. SPSS® 22.0 software was used for the analysis.

The present study was approved by the Research Ethics Committee (4.865.026) and all participants signed the Informed Consent Form.

## Results

**Figure 1:**
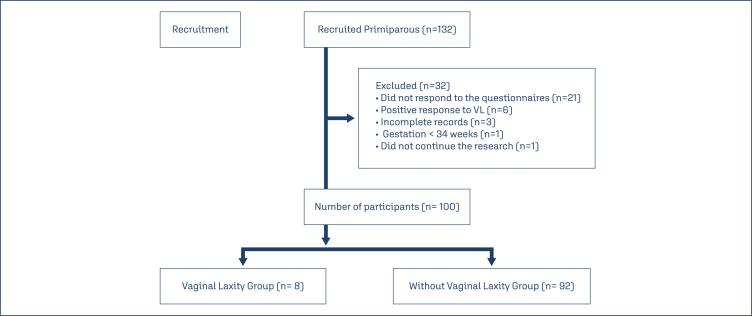
Flowchart of primiparous women recruited for the study

During the period from July 2021 to January 2023, 132 primiparous participants were recruited for data analysis. Thirty-two participants were excluded (21 did not respond to the questionnaires; six responded positively to VL; three were due to incomplete medical records; one had a gestation of less than 34 weeks and one did not want to continue in the research). Of the 100 participants with complete data included in the final analysis, eight reported VL and 92 denied this symptom (Figure 1).

The prevalence of VL after childbirth in the present study was 8%. The sociodemographic characteristics of the participants are shown in table 1. The average age in participants with and without VL were 24.6 ± 6.3 and 26.1 ± 6.0, and the average weight of the newborns was 3020g ± 594.32 and 3165gr ± 459.9, respectively. Regarding obstetric clinical data, only shoulder dystocia and coital incontinence were statistically different between participants, despite the single occurrence in the VL group. Stress urinary incontinence (SUI) was more frequent in participants with VL (62.50%) compared to participants without VL (45.65%). In the same evaluation period, 100% of participants with VL were sexually active compared with only 65.22% of participants without VL (Table 1).

**Table 1:** Sociodemographic, clinical and obstetric characteristics of the included participants

Variables		Vaginal laxity (n=8) n%	Without vaginal laxity (n=92) n%	p-value
Age (mean ± SD)		24.6 ± 6,3	26.1 ± 6,0	0.437
Education				0.296
	Primary	1(12.50)	2(2.17)	
	Secondary	6(75)	76(82.61)	
	Tertiary	1(12.0)	14(15.22)	
Body Mass Index				0.651
	Normal	1(12.50)	23(25)	
	Overweight	3(37.50)	35(38.04)	
	Obesity	4(55.50)	34(36.96)	
Gestation				0.763
	Single gestation	8(100)	89(96.74)	
	Twin gestation	0	3 (3.26)	
Type of birth				0.062
	Vaginal Delivery	7(87,50)	49(53.26)	
	C-section	1(1)	43(46.74)	
Gestational age / Mean ± SD		38.5 ± 1.9	38.4 ± 1.4	0.768
Newborn weight / Mean ± SD*		3,020 ± 594.2	3,165 ± 459.9	0.237
Newborn weight / Grams*				0.653
	< 2500	1(12.50)	12(13.05)	
	2500 – 3499	6(75)	62(67.39)	
	3500 – 4000	0	16(17.39)	
	> 4000	0	2(2.17)	
Forceps		0	3(3.26)	0.598
Vacuum extraction		0	1(1.09)	0.764
Position during the second stage of labour*				0.473
	Not applied (C-section)	1(12.50)	43(46.74)	
	Lithotomy	1(12.50)	12(13.05)	
	Sitting position	2(25)	15(16.30)	
	Squatting position	3(37.50)	20(21.74)	
Hands-and-knees position		-	2(2.17)	
Duration of the second stage of labour (mean ± SD)		39.4 ± 40.6	60.9 ± 49.4	0.343
Oxytocin *		3(37.50)	46(50)	0.498
Spinal anaesthesia		1(12.50)	42(45.65)	0.070
Epidural anaesthesia		4(50)	38(41.30)	0.625
Episiotomy		2(25)	6(6.52)	0.070
Perineal laceration				0.383
	0	2(25)	48(52.17)	
	1	3(37.50)	20(21.74)	
	2	3(37.50)	20(21.74)	
	3	0	4(4.35)	
Shoulder dystocia		1(12.50)	1(1.09)	0.029
Stress urinary incontinence		5(62.50)	42(45.65)	0.357
Urge urinary incontinence		1(12.50)	15(16.30)	0.760
Urgency		3(37.50)	42(45.65)	0.661
Nocturia		7(87.50)	88(95.65)	0.321
Constipation		6(75)	65(70.65)	0.675
Anal incontinence		1(12.50)	7(7.61)	0.640
Dyspareunia		2(25)	17(18.48)	0.613
Sexual activity		8(100)	60(65.22)	0.988
Coital incontinence		1(12.50)	2(2.17)	<0.001

* Data not reported in one or both groups. Mean ± SD: Standard Deviation. Fisher and Chi-square test. Mann-Whitney test.

Urinary, bowel and sexual data were collected in two periods, both at initial recruitment and six months postpartum. The majority of urinary symptoms investigated improved six-months after birth, with statistical significance between the two periods. Anal incontinence, coital incontinence and dyspareunia worsened six-months postpartum, but there was no statistically significant difference (Table 2).

**Table 2:** Comparison between Clinical variables and questionnaire assessment periods

Variables		Initial recruitment (n)	6 months postpartum (n)	p-value
Urinary symptoms				
Stress urinary incontinence		47	10	<0.001
Use of absorbent pads		14	12	0.824
Urgency		45	10	<0.001
Urge urinary incontinence		16	9	0.144
Urinary frequency				<0.001
	< 8x	20	28	
	> 8x	80	72	
Nocturia		95	67	<0.001
Nocturnal enuresis		7.1	4	0.508
Dysuria		5	5	0.999
Suprapubic pain with repletion		26	9	0.002
Straining to urinate		10	11	0.999
Feeling of incomplete emptying		49	22	<0.001
Intermittent stream		20	15	0.424
History of urinary tract infection		33	11	<0.001
Bowel symptoms				
Bowel habit				0.052
	Regular	19	30	
	Constipation	81	70	
Anal incontinence		8	18	0.064
Sexual symptoms				
Coital incontinence/orgasm		0	3	0.500
Dyspareunia		19	21	0.839
Sexual activity				0.230
	No	38	31	
	Yes	62	69	

McNemar test

The total score of the ICIQ-SF questionnaire decreased significantly (p<0.001) six months after birth when compared to initial recruitment, indicating an improvement in the symptoms of UI and the impact on quality of life. In the vaginal symptoms score of the ICIQ-VS questionnaire, a statistically significant difference was found for the same period (p=0.002). The other ICIQ-VS scores for sexual issues (p=0.117) and quality of life (p=0.204) also decreased, suggesting an improvement in symptoms, but did not reach a statistically significant difference. The total score of the FSDS-R questionnaire (p=0.722) was higher six-months after birth, signalling a worsening of sexual distress during this period, but there was no statistically significant difference (Table 3).

**Table 3:** Comparison between questionnaire scores and questionnaire assessment periods

Questionnaires		Initial recruitment (mean ± SD)	6 months postpartum (mean ± SD)	p-value
ICIQ-SF		3.18 ± 3.89	1.41 ± 3.76	<0.001
ICIQ-VS				
	Vaginal Symptoms	6.61 ± 4.87	4.99 ± 5.73	0.002
	Sexual Matters	6.03 ±11.22	5.02 ± 14.74	0.117
	Quality of life	2.02 ± 2.76	1.71 ± 2.94	0.204
FSDS-R		4.11 ± 6.60	5.35 ± 10.77	0.722

Mean ± SD: Standard Deviation; ICIQ-VS: *International Consultation on Incontinence Questionnaire Vaginal Symptoms*; FSDS-R: *Female Sexual Distress Scale Revised*; ICIQ-SF: *International Consultation on Incontinence Questionnaire* – *Short Form*

The clinical data SUI, urgency urinary incontinence (UUI), coital incontinence and intestinal constipation were associated with VL symptoms in the univariate analysis. However, only UUI and coital incontinence remained associated with VL in the multivariate-analysis (Table 4). Regarding the questionnaire scores, the ICIQ-VS, the FSDS-R and the ICIQ-SF increased the risk of VL in the participants by one, in the univariate analysis. However, only the ICIQ-VS (vaginal symptoms domain) and the ICIQ-SF remained associated with VL in the multivariate-analysis, OR 1.32 and OR 1.25, respectively.

**Table 4:** Association between clinical variables and complaints of vaginal laxity

Variables	Univariate analysis OR (95%CI)	p-value	Multivariate analysis OR (95%CI)	p-value
Childbirth				
Caesarean section	0.14 (0.02 – 1.16)	0.069		
Perineal laceration				
Grade II	5.05 (0.85 –29.92)	0.074		
Second Stage of labour				
Sitting position	9.21 (0.89 –95.86)	0.063		
Stress urinary incontinence	11.33 (2.40 – 53.60)	0.002		
Urge urinary incontinence	7.08 (1.41 – 35.59)	0.017	10.50 (1.90 – 58.10)	0.007
Coital incontinence	25.71 (2,07 – 319.80)	0.012	42.00 (3.11 – 566.38)	0.005
Constipation	5.58 (1.29 – 24.10)	0.021		
ICIQ-VS				
Vaginal symptoms	1.31 (1.11 – 1.55)	0.001	1.32 (1.05 – 1.66)	0.015
Sexual matters	1.04 (1.00 – 1.09)	0.031		
Quality of life	1.31 (1.08 – 1.60)	0.007		
FSDS-R	1.05 (1.00 – 1.09)	0.036		
ICIQ-SF	1.33 (1.14 – 1.56)	0.001	1.25 (1.02 – 1.54)	0.028

Logistic Regression; OR: Odds Ratio; CI: Confidence Interval; ICIQ-VS: *International Consultation on Incontinence Questionnaire Vaginal Symptoms*; FSDS-R: *Female Sexual Distress Scale Revised*; ICIQ-SF: *International Consultation on Incontinence Questionnaire – Short Form*

## Discussion

VL remains little investigated in the literature, being a complaint that negatively affects women's sexual function and quality of life.^(12,13)^ In the present study, the prevalence of VL in women after their first birth was 8%. The symptom of UI and the impact on quality of life assessed by the ICIQ-SF, as well as the vaginal symptoms assessed by the ICIQ-VS improved six-months after delivery. In the univariate analysis, the scores of the ICIQ-SF, ICIQ-VS and FSDS-R questionnaires, in addition to clinical data such as SUI, UUI, coital incontinence and intestinal constipation six-months postpartum were associated with VL symptoms. However, only the ICIQ-VS vaginal symptoms domain, the ICIQ-SF score, UUI and coital incontinence maintained an association with the VL symptom in the multivariate-analysis.

Regarding clinical/obstetric data, only shoulder dystocia (p=0.029) and coital incontinence (p<0.001) were statistically different between the participants. Packet et al.,^(20)^ identified that shoulder dystocia was one of the predictive factors for obstetric injury to the anal sphincter in primiparous women. This type of injury could have some implications for the integrity of the PFM and be related to VL. However, we found no association between VL and shoulder dystocia and coital incontinence.^(20)^ Coital incontinence appears to be a multifactorial, underreported symptom, associated with the severity of SUI and with a negative effect on quality of life and female sexual function.^(21)^

Most urinary symptoms and ICIQ-SF scores improved six-months postpartum. This can be explained by the physiological changes during pregnancy that imply a greater occurrence of urinary dysfunction during this period compared to postpartum.^(22–24)^

It is worth mentioning that the presence of urinary incontinence before pregnancy is the predictive factor for UI during pregnancy, in addition to increasing the chances of developing UI in the postpartum period by up to three times.^(25,26)^ However, we did not evaluate urinary complaints in the period before pregnancy. Leeman et al.^(27)^ found a higher number of reports of UI in the third trimester of pregnancy (74.1%) compared to six-months postpartum in primiparous women (59.8%) and van Delft et al.^(28)^ revealed fewer UI symptoms three months postpartum (64%) compared to the third gestational trimester (74%). Stress urinary incontinence was the most prevalent type of UI in the present study (47%), similar to other studies.^(22,24,25,28)^ The ICIQ-SF score and the UUI symptom remained associated with the VL symptom in our multivariate-analysis, similar to the study by Campbell et al.^(5)^ and Pereira et al.^(29)^ The findings of Dietz et al.^(1)^ and Talab et al.^(4)^ contrast with ours, since VL was not associated with any type of UI investigated. To date, we have not found studies that investigated the association between coital incontinence and the symptom of VL. Although the multivariate-analysis was statistically significant for these variables, the small number of VL cases in this sample does not allow these findings to be extended to all women with VL.

Obstetric data such as type of delivery (caesarean section), laceration (grade 2) and position of the expulsion (sitting), despite not having reached statistical significance, presented values close to 5% and clinical relevance for our research. Similarly to the study by Talab et al.,^(4)^ caesarean section (p=0.069) when compared to vaginal delivery, appeared to be a protective factor against the symptom of VL. Perineal trauma is common during vaginal birth, reaching a prevalence of 85%, with primiparity being a risk factor for severe lacerations (grades 3 and 4).^(30)^ However, only four participants without VL presented grade III tears, with grade I and II tears (mild lacerations) being more frequent in our sample. In participants with VL, grade II laceration had a significance level close to 5% (p=0.074). This may suggest that damage to the integrity of the PFM, caused by grade II lacerations, contributes to the presence of the VL symptom after the first birth. However, we did not objectively assess the presence or absence of injury to the levator ani muscle.

The position during the expulsion period that came closest to the significance value of p=0.05 was the sitting position (p=0.063). In the study by Reis et al.,^(31)^ the sitting position was the most used and no association was established between this position and the symptom of VL.

The presence of vaginal symptoms assessed by the ICIQ-VS in this study significantly decreased six-months after birth but increased the chance of developing VL by one. The study by McDonald et al.^(32)^ contrasts with our findings due to the increase in vaginal symptoms in primiparous women in the postpartum period due to lack of lubrication (29.6% during pregnancy and 48.2% six-months postpartum); vaginal tightness (21.2% during pregnancy and 34.1% six-months postpartum). O’Malley et al.^(33)^ evaluated vaginal symptoms such as vaginal tightness, lubrication and VL in primiparous women at the beginning of pregnancy and six-months after birth and also found worsening in the second stage. However, the authors did not use a validated questionnaire. Another study that differs from our results is by Abdool et al.,^(34)^ who evaluated 83 primiparous South African women in the third trimester and three to six-months after giving birth to determine the impact of giving birth on PFM. ICIQ-VS vaginal symptom scores for VL, dry vagina, interference with sexual life and quality of life were significantly higher in the postpartum period.

Despite not reaching a statistically significant difference between participants, the total FSDS-R score worsened six-months after birth. First-time women can take three to eighteen months to improve pelvic floor disorders that negatively affect sexual health.^(35)^ In the study by Witting et al.,^(36)^ primiparous women had lower sexual satisfaction and greater intensity of pain during sexual intercourse when compared to multiparous women. Pereira et al.^(2)^ found higher scores on the FSDS-R in women with VL when compared to those without VL. In another study, the symptom of VL was predictive of sexual distress assessed by the FSDS-R in women with and without breast cancer.^(6)^

Approximately 31% of participants had not resumed sexual activity six months after delivery. The low prevalence of VL in primiparous women in our study may have been influenced by this rate, since the symptom of VL is perceived during sexual intercourse.^(1,4,5)^ However, to date, we have not found any study in the literature that evaluated VL in a population exclusively of primiparous women as a primary outcome, based on a validated questionnaire and its associations with sexual, urinary, intestinal symptoms and obstetric data.

This study has limitations. First, participants were not assessed for the presence of symptoms prior to pregnancy. Additionally, the maternity hospital where the research was conducted serves multiple cities within the state, making it unfeasible to contact participants during the gestational period; thus, contact was only possible upon admission for delivery. Furthermore, key factors such as the impact of breastfeeding on sexual function, the nature of sexual activity, and the reasons for the absence of sexual relations were not evaluated. The diagnosis of VL was based on self-reported data obtained through direct questioning and a validated questionnaire. However, no physical examinations or ultrasound assessments were performed, which may have affected diagnostic accuracy. Moreover, the retrospective nature of the question regarding the presence or absence of VL during pregnancy at the time of initial recruitment introduces a potential recall bias. Additionally, the study design does not allow for the establishment of causal relationships. Another limitation is the small number of participants with VL (n=8), which restricts the generalizability of the findings and increases the risk of Type II errors. Therefore, the results should be interpreted with caution. Future studies should aim for a larger sample size and a longitudinal design to provide a better understanding of the role of pregnancy and childbirth in the development of VL.

Despite these limitations, the strengths of our study include the assessment of the prevalence of an underreported symptom, specifically among primiparous women without a history of this complaint, thereby mitigating the confounding effects associated with parity. Additionally, the study successfully identified associations between VL and symptoms of pelvic floor dysfunction—specifically urinary, vaginal, and sexual symptoms—using validated questionnaires.

## Conclusion

The prevalence of VL, in this sample of primiparous women, was lower than that reported in other studies and showed an association with the occurrence of vaginal symptoms, urgency urinary incontinence and coital incontinence, six-months postpartum.
